# Clinical and Imaging-Based Prognostic Models for Recurrence and Local Tumor Progression Following Thermal Ablation of Hepatocellular Carcinoma: A Systematic Review

**DOI:** 10.3390/cancers17162656

**Published:** 2025-08-14

**Authors:** Coosje A. M. Verhagen, Faeze Gholamiankhah, Emma C. M. Buijsman, Alexander Broersen, Gonnie C. M. van Erp, Ariadne L. van der Velden, Hossein Rahmani, Christiaan van der Leij, Ralph Brecheisen, Rodolfo Lanocita, Jouke Dijkstra, Mark C. Burgmans

**Affiliations:** 1 Department of Radiology, Leiden University Medical Center (LUMC), 2333 ZA Leiden, The Netherlands; c.a.m.verhagen@lumc.nl (C.A.M.V.); f.gholamiankhah@lumc.nl (F.G.); e.c.m.buijsman@lumc.nl (E.C.M.B.); a.broersen@lumc.nl (A.B.); g.c.m.van_erp@lumc.nl (G.C.M.v.E.); j.dijkstra@lumc.nl (J.D.); 2 Department of Radiology and Nuclear Medicine, Maastricht University Medical Center+, 6229 HX Maastricht, The Netherlands; lisa.van.der.velden@mumc.nl (A.L.v.d.V.); hossein.rahmani@maastrichtuniversity.nl (H.R.); christiaan.vander.leij@mumc.nl (C.v.d.L.); 3 GROW, Research Institute for Oncology and Reproduction, Maastricht University, 6211 LK Maastricht, The Netherlands; 4 Department of Surgery, Maastricht University Medical Center+, 6229 HX Maastricht, The Netherlands; r.brecheisen@maastrichtuniversity.nl; 5 NUTRIM School of Nutrition and Translational Research in Metabolism, Maastricht University, 6211 LK Maastricht, The Netherlands; 6 Department of Radiology, Foundation IRCCS Istituto Nazionale Tumori, 20133 Milan, Italy; rodolfo.lanocita@institutotumori.mi.it

**Keywords:** systematic review, hepatocellular carcinoma, thermal ablation, prognostic models, recurrence, local tumor progression

## Abstract

**Background:** Early detection of patients at high risk for recurrence or local tumor progression (LTP) following thermal ablation of hepatocellular carcinoma (HCC) is essential for treatment selection and individualized follow-up. This systematic review aims to assess and compare the performance of prognostic models predicting recurrence or LTP in patients with HCC treated with thermal ablation. **Methods:** PubMed, Web of Science, Cochrane, and Embase were searched for studies developing models to predict recurrence after thermal ablation in treatment-naïve HCC patients, using imaging and clinical data with reported test set performance. Risk of bias and applicability were assessed by the Prediction model Risk of Bias Assessment Tool. Data on model performance, feature extraction and modeling technique was collected. **Results:** In total, 16 studies comprising 39 prognostic models were included, all developed using retrospective data from China or Korea. Outcomes included recurrence-free survival, (intrahepatic) early recurrence, LTP, late recurrence and aggressive intrasegmental recurrence. Predictive parameters varied across models addressing identical outcomes. Outcome definitions also differed. Nine models were externally validated. Most studies had a high risk of bias due to methodological limitations. **Conclusions:** Variability in model development methodology and type of predictors was found. Models that integrated multiple types of predictors consistently outperformed those relying on one type. To advance predictive tools toward clinical implementation, future research should prioritize standardized outcome definitions, external testing, and transparent reporting. Until these challenges are addressed, current evaluated models should be regarded as promising but preliminary tools.

## Simple Summary

This review examined tools designed to predict whether liver cancer will return after a heat-based locoregional treatment, which is called thermal ablation. For development of the prediction models, a wide range of techniques were used to identify predictive factors. Predictive models that combined different types of information, such as clinical- and medical imaging data, performed better than those relying on a single type. It was found that studies often used different definitions for outcomes and lacked proper testing methods, making results hard to compare. While some models show promising performance, they are not ready for use in clinical practice yet, due to methodological shortcomings. Future research should therefore focus on consistent definitions, external testing, and transparent development.

## 1. Introduction

Tumor recurrence is a key determinant in the long-term prognosis of patients undergoing thermal ablation (TA) in the management of HCC. Early diagnosis of recurrence enhances the likelihood of curative retreatment and improved oncological outcomes. As such, accurately identifying patients at high risk is essential for guiding treatment selection and tailoring postprocedural follow-up. Prognostic assessment in HCC is complex due to interindividual differences and heterogenous carcinogenic behavior, resulting in varied treatment responses among patients classified within the same Barcelona Clinic Liver Cancer (BCLC) stage [1,2,3,4]. This suggests that one-size-fits-all prognostic approaches may underperform those that are tailored for individualized use. A range of inputs can be integrated for outcome prediction, such as clinical parameters, conventional imaging features, or more sophisticated imaging data such as radiomics and deep learning (DL) derived features. Artificial intelligence (AI) has gained increasing interest because of its capability to identify and extract predictive factors that may be imperceptible to human observation [5]. Radiomics, as an advanced image analysis tool, transforms images into a high-dimensional feature space by extracting quantitative features [6]. Unlike conventional statistical models, AI based approaches can integrate clinical and imaging data in an end-to-end manner, enabling automated feature extraction and outcome prediction [7].

Several literature reviews on prognostic models for HCC patients have been published; however, they focus on other treatments such as systemic therapy or surgical resection [8,9], include both primary and secondary liver tumors, or exclusively assess AI-based models [10,11]. This systematic review aims to evaluate and compare the performance of prognostic models developed to predict recurrence in patients with HCC treated with TA, considering both statistical and AI-based models.

## 2. Materials and Methods

### 2.1. Literature Search

The protocol was prospectively published in the International Prospective Register of Systematic Review (PROSPERO) with ID: CRD42024503425. This systematic review is compliant with the Preferred Reporting Items for Systematic Reviews and Meta-Analysis (PRISMA) 2020 guidelines [12]. PubMed, Web of Science, Cochrane and Embase were searched from their inception until 14 March 2025. The search strategy was composed by an experienced librarian from the Walaeus Library (Leiden University Medical Center). Syntax included synonyms for (a) Thermal ablation (b) Hepatocellular Carcinoma (c) Conventional or deep learning based predictive models. Details of the search strategy are provided in Table A1 in the Appendix A.

### 2.2. Eligibility Criteria

Qualified studies were those that integrated advanced imaging features (those other than “tumor number” and “maximal tumor diameter”) to predict recurrence following TA of HCC. Exclusion criteria included ex vivo or animal-based models, pretreated patient cohorts, patients treated with combined strategies (e.g., transarterial chemoembolization and TA or adjuvant chemotherapy following TA), models based on non-routine clinical data (e.g., genomic sequencing), publications not written in English, unavailability of full text, and lack of reported model performance on test sets. Two reviewers (F.G., C.V.) independently screened the articles identified by the search. Eligibility was considered following reviewing the full manuscript of the potential study. Data was extracted by F.G. and C.V independently. In the event of disagreement between the two primary reviewers, independent reviewers (J.D., M.C.B., A.B.) were consulted to reach consensus.

### 2.3. Methodological and Reporting Quality

Data were extracted using the Checklist for Critical Appraisal and Data Extraction for Systematic Reviews of Prediction Modelling Studies (CHARMS) [13]. In addition, data was extracted on ablation and imaging modality, feature selection and model development technique. The Prediction model Risk of Bias Assessment Tool (PROBAST) was used to assess the risk of bias (ROB) for each model and evaluate the applicability of the studies included [14]. ROB and quality assessment was performed by FG and CV independently. A consensus on discrepancies was obtained by discussing them with three independent parties (M.C.B., J.D., A.B.). Studies were grouped and compared according to each specific outcome. Additionally, studies were categorized based on the type of validation reported (internal vs. external) and the combination of predictor types used. For each synthesis, only studies that reported the relevant outcome measure were included. When a study was developed or validated using more than one prognostic model, each model was analyzed based on the combination of predictors used. If multiple models used the same type of variables but in varying combinations (e.g., different combinations of radiomics features), the best-performing model was selected. Discriminative performance was assessed using Area Under the Receiver Operating Characteristic curve (AUC-ROC) and Concordance-index (C-index). Calibration was evaluated using calibration slope, intercept, or the Hosmer–Lemeshow test. Clinical utility was assessed through decision curve analysis (DCA). For studies with risk group stratification, Kaplan–Meier curves were used to compare survival. The extracted data were visualized using forest plots, accompanied by tables highlighting the key elements.

## 3. Results

### 3.1. Study Selection

**Figure 1:**
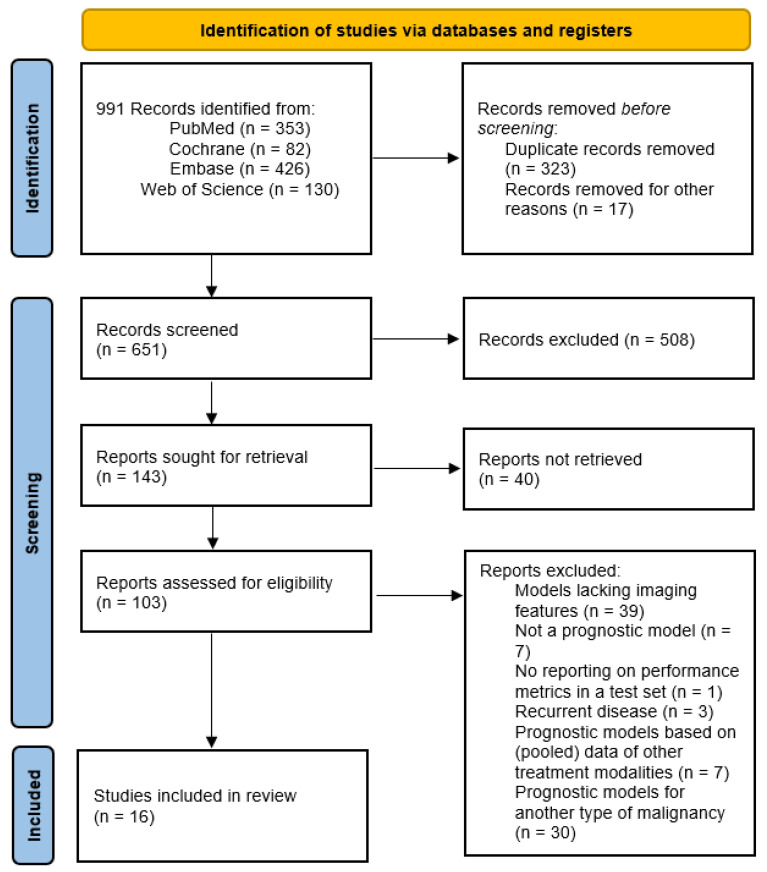
Flowchart of the search strategy and study selection in accordance with the Preferred Reporting Items for Systematic Reviews and Meta-Analyses (PRISMA) guidelines.

The database search yielded 991 titles. Endnote was utilized to manage references, remove duplicates and non-English studies (*n* = 323 and *n* = 17, respectively). Then, 651 records were screened, of which 103 potential articles underwent a full comprehensive review. Subsequently, 16 studies met the inclusion criteria and were included. The overall selection process is illustrated in Figure 1.

### 3.2. Study Characteristics

Table 1 summarizes key features of the included articles, focusing on tumor characteristics and outcome definition. Table 2 outlines the key characteristics of the included models, focusing on datasets and evaluation. Eleven studies developed more than one model, resulting in a total of 39 models. Of these, 2 models used only clinical predictors, 18 relied solely on imaging predictors, and 19 incorporated both imaging and clinical predictors. All prognostic models were developed between 2019 and 2025 and based on retrospectively collected data from China or Korea. Sample sizes ranged from 58 to 513 individuals. Three studies were multicenter based [15,16,17]. Tumor-related inclusion criteria often set a maximum threshold of a ≤3 cm diameter for single tumors [16,18,19], or a combined diameter of ≤3 cm for multiple tumors [18]. Larger tumors up to 5 cm were included in 7 studies [15,20,21,22,23]. Three studies did not specify their tumor-related criteria other than it being HCC [17,24,25]. Radiofrequency ablation (RFA) was used in six studies [18,19,20,21,24,26,27], microwave ablation (MWA) in two studies [17,27,28], and both RFA and MWA were used in 7 studies [15,16,22,23,25,29].

### 3.3. Prognostic Model Outcome

Except for Ma et al. [23] and Wu et al. [30], all studies developed models with a single clinical outcome. Early recurrence (ER), defined as recurrence within 24 months following the ablation, was the most common. The definition of tumor progression or recurrence varied across studies (Table 1).

### 3.4. Prognostic Factors

#### 3.4.1. Clinical Predictors

**Figure 2:**
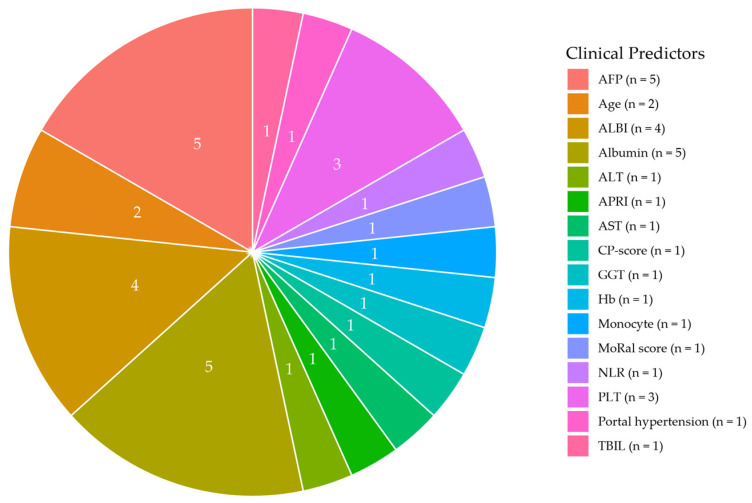
Clinical variables incorporated by the prediction models, stratified by count. Abbreviations: AFP, Alpha fetoprotein; ALBI, Albumin-bilirubin grade; ALT, Alanine Transaminase; APRI, (AST/40) × 100/PLT; AST, Aspartate aminotransferase; CP-score, Child-Pugh-score; GGT, Gamma-glutamyl transferase; Hb, Hemoglobin; MoRal, Model Of Recurrence After Liver transplant; NLR, Neutrophil-to-lymphocyte ratio; PLT, Platelet count; TBIL, Total bilirubin. Note: For studies that developed multiple models including clinical variables, each clinical variable is counted once.

All clinical prognostic variables were measured preprocedural, except for the clinical model and the clinical-texture model developed by Li et al. [18], which incorporated the albumin-bilirubin (ALBI) grade sampled two to four weeks after the procedure. Albumin was the most commonly included clinical factor, appearing either as a continuous or binary variable [15,26,29], as part of the ALBI grade [18,19,30] or Child-Pugh score [25] (Figure 2). The second most common clinical factor was the alpha fetoprotein (AFP) concentration, which was included in five studies [21,22,24,25,29], either as a binary variable or categorical variable. Cutoffs for the (binary) categories varied. There was no uniform set of clinical variables associated with each outcome. A comparison of the models for ER and LR developed by Ma et al. [23] revealed that portal hypertension, alanine transaminase, and hemoglobin levels were independent predictors for LR but not for ER, indicating that distinct predictors may be associated with different recurrence patterns. In contrast, the clinical predictors for ER and LR did not differ in the models developed by Wu et al. [30].

Among the studies included in this review, 7 used data from both RFA and MWA procedures. Of these, 4 papers considered the ablation modality as a candidate predictor during feature selection. Zhang L et al. [29], Ma et al. [23] and Huang et al. [25] reported no significant *p*-value for the ablation modality in univariate analysis (*p* 0.88, 0.34 and 0.61, respectively). Li FY et al. [16] reported a significant *p*-value (*p* = 0.076) for the ablation modality in univariate analysis; however, it was not significant in multivariate analysis (*p* = 0.134) and therefore excluded from the final prediction model.

#### 3.4.2. Imaging-Based Predictors

The models integrating imaging-related predictors are listed in Table 3. Most models solely used preprocedural based predictors, whereas 5 models used both pre- and postprocedural imaging predictors [16,17,28], and 3 models only used postprocedural predictors [18]. Imaging-based predictors were categorized by the complexity of feature extraction into radiological, radiomics, and DL-based features. The most frequently used radiological features were number of tumors [15,17,22,24,29,30] and tumor size [16,20,28,29,30]. Tumor location was included as a variable in two models. One defined location based on proximity to abutting vessels [17], while the other categorized “high risk location” as location within 0.5 cm of the intrahepatic large vessels or the surrounding organs or structures [25]. In models involving postprocedural imaging, the ablation margin (AM) was frequently used [16,17,21]. Various techniques were utilized for the AM measurements: Li FY et al. [16] measured the AM as the shortest distance from the outer margin of the ablation zone, plane-by-plane (i.e., 2-dimensional), whereas Chen et al. [17] manually delineated the ablation zone and tumor, enabling a three-dimensional (3D) reconstruction. Some studies [18,19,21,25,28] calculated features from the Liver Imaging Reporting and Data System (LI-RADS) [31].

Radiomics features were included in 17 models [15,17,23,26,27,29,30]. For radiomics feature extraction, regions-of-interest or volumes-of-interest were defined around the tumor [15,18,20,23,25,26,27,29] or ablation zone [17,18] with some studies including the peritumoral area or adjacent normal liver parenchyma [17,20,23,29]. Table 3 presents the main category of included radiomics features and the number of features from each category, Table A2 provides a detailed list of the names of all used features.

Another group of explored factors includes DL-based features, automatically extracted from imaging. Some studies used DL models solely for feature extraction, while others employed DL end-to-end for both feature selection and outcome prediction (Section 3.5). Convolutional neural networks (CNNs) were predominantly used for feature extraction, either through pre-trained models [17] or by training models on the study-specific dataset [15,20,30]. Ma et al. [23] developed models combining CNNs and recurrent neural networks to extract spatiotemporal features from arterial and delayed portal venous phases of contrast-enhanced ultrasound (CEUS) images.

### 3.5. Feature Selection Techniques and Model Development

Detailed information on the prognostic features selected for model development is provided in Table 3. Lasso regression was predominantly employed for radiomics feature selection. While some studies used a single technique for feature selection [12,18,27], others explored a combination of two or three techniques [15,25,26]. Additionally, in some models, relevant features were automatically extracted and selected within a DL framework [15,20,23,30]. In prognostic models incorporating both clinical and imaging features, one approach was to integrate features selected separately and used in individual models [17,18,23,25,29]. Alternatively, candidate features were merged followed by a feature selection technique [15,26].

Some studies aimed to develop a single model incorporating all selected predictors [19,21,22,24,28,30], while others created separate models for different feature categories (i.e., clinical, radiological, radiomics, DL-based features) as well as a combined model [9,15,17,20,21,23,25,27,28,29] and compared their performance. This could involve using different model types for each feature category [23], or the same model for all [17]. In combined models integrating all feature types, a nomogram was typically developed using multivariate (MV) logistic regression or Cox regression [9,19,20,21,22,23,24,25,28]. Only three studies used AI-based models [15,17,23].

### 3.6. Model Performance

**Figure 3:**
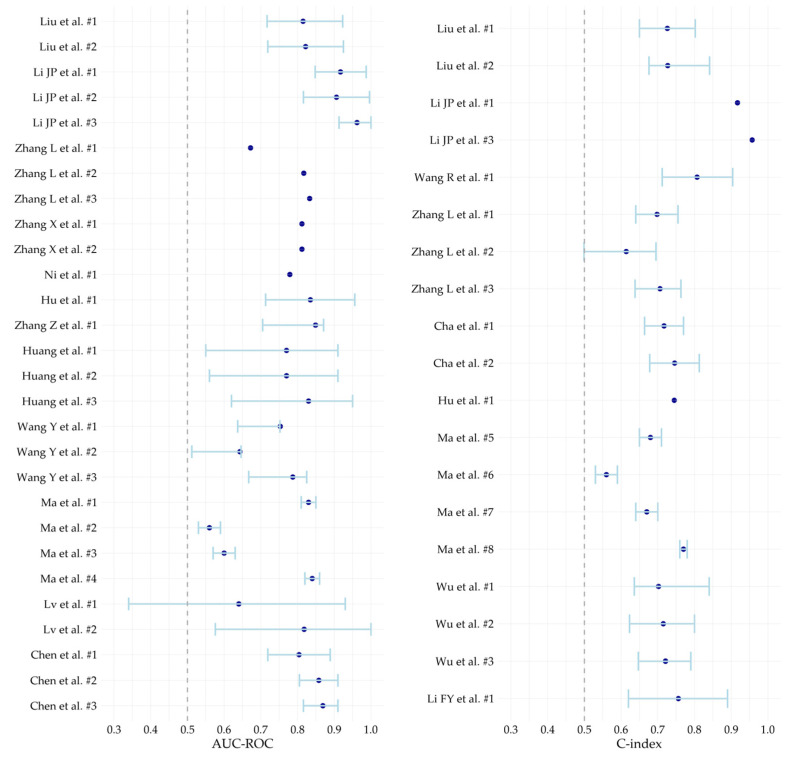
Forest plot of discrimination metrics [15,16,17,18,19,20,21,22,23,24,25,26,27,28,29,30]. Abbreviations: AUC-ROC, Area Under the Receiver Operating Characteristic curve; C-index, Concordance index.

Table 4 summarizes the characteristics of the models included, detailing predictor types, and performance metrics. Additionally, forest plots of the C-indexes and AUC-ROCs are presented in Figure 3. Among the models that prognosticated recurrence free survival (RFS), the C-index ranged from 0.61 to 0.96 [18,20]. The clinical texture model by Li JP et al. [18], incorporating clinical and radiomics features, demonstrated excellent discriminative performance, with a C-index of 0.96 and an AUC-ROC of 0.96 (95% Confidence Interval (CI): 0.91–1.00) at 24 months, alongside good calibration (HL *p* = 0.72) and favorable net benefit under different probability thresholds in the DCA. Yet, the training cohort merely included 63 patients, and bootstrap resampling was used to create a cohort for internal testing, which increased the likelihood of overoptimistic performance estimates.

For the prediction of ER, six models were externally tested [15,25]. The nomogram developed by Huang et al. [25] was the best performing model with an AUC-ROC of 0.83 (95% CI: 0.62–0.95), good calibration (HL *p* = 0.40), and superior net benefit in the DCA across different threshold probabilities. The small external test cohort (*n* = 25) likely contributed to the wide CI, introducing a degree of uncertainty concerning the generalizability in a larger external cohort. Wang Y et al. [15] reported on an externally tested model with comparable discriminative performance for intrahepatic ER, which was tested on a larger test cohort (*n* = 116), achieving an AUC-ROC of 0.79 (95% CI: 0.67–0.82) and favorable net benefit in DCA; however, calibration metrics were lacking which limits the reliability of the reported discrimination performance.

Lastly, five models were developed for LR prognostication, all internally tested [23,30]. The best performing model was developed by Ma et al. [23], with a C-index of 0.77 (95% CI: 0.76–0.78), a positive net clinical benefit, and good calibration. However, the calibration assessment was based on visual interpretation, since quantitative metrics for this were lacking.

For LTP prediction, the DL-radiomics based model using both preprocedural and postprocedural signatures multiparametric-MRI from Chen et al. [17], demonstrated excellent discriminative performance (C-index 0.87, 95% CI 0.81–0.91), good calibration, and significant OS differences between low- and high-risk groups in two external cohorts (*n* = 135, *p* = 0.00039; *n* = 93, *p* = 0.0021). Li FY et al. [16] was the only study that developed and internally tested a prognostic model for LTP free survival (LTPFS), which performed moderately with an AUC-ROC of 0.76 (0.62–0.89) and good calibration.

Various strategies were implemented to mitigate overfitting: cross validation [15,18,23,29], bootstrap resampling [18,19,29], temporal normalization [20] and data augmentation [30]. Despite this, signs of overfitting (reflected by a decline in performance metrics from the training to the test cohort) were still evident at different levels: either during internal validation [23,26,27,29,32], while in others it became evident only during external testing [15].

### 3.7. Risk of Bias Assessment

**Figure 4:**
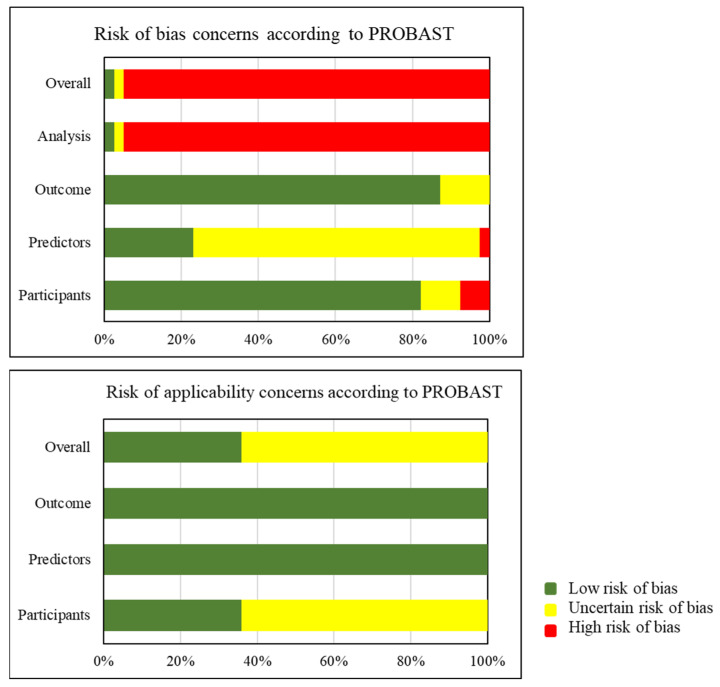
Risk of bias and applicability according to PROBAST. Abbreviations: PROBAST, Prediction model Risk of Bias Assessment Tool.

The results of the PROBAST assessment are shown in Figure 4. A detailed overview of the risks per study is provided in Table A3 of the Appendix A. Overall, the majority of developed models qualify for high ROB, except for model 1 by Liu et al. [20], and model 2 by Wang Y et al. [15]. In the participants domain, ROB was mainly due to the setting of data collection: retrospective, single center, or missing details on the handling of missing data. Inconclusive ROB was found in 4 models due to missing exclusion details (34), and discrepancies in reported numbers of exclusions [27]. In the predictor domain, high ROB was found in one model [15] due to inconsistent predictor definitions across institutions, while all models with unclear ROB lacked information on blinding during predictor assessment [9,15]. Most models qualified for low ROB in the outcome domain, while five models were classified as unclear ROB due to incomplete data on follow-up [15,27]. All models had high ROB in the analysis domain, except for two DL models [15,20], due to low event per variable (EPV < 20), excluding patients after inclusion [22,23], dichotomization of continuous predictors [9,15,17,21,22,24,28,29,30], and/or suboptimal variable selection [9,15,17,19,21,22,24,25,26,28,29,30]. Additional issues included incomplete reporting of radiomics feature selection [30], and lack of transparency regarding predictor weights and data complexities [15,17,21,22,24,25,26,28,30]. Lastly, calibration is most reliably assessed using the calibration slope (ideal = 1; <1 indicates overfitting, >1 underfitting) and the calibration intercept (ideal = 0; <0 suggests overestimation, >0 underestimation). However, most studies included in this review evaluated it by the HL goodness-of-fit test [18,20,22,25], despite its low statistical power (Table 4) [33]. The applicability of most studies in the participant domain was unclear due to the predominance of hepatitis B virus related HCC in Asian cohorts. Models that did not consider underlying liver disease as a predictor were assessed as having unclear applicability [15,16,17,20,22,23,25,26,27,28,29,30]. No high concerns were noted in other domains.

## 4. Discussion

This review identified several techniques and modeling strategies to predict recurrence following TA of HCC in treatment of naive patients. It demonstrates that models that incorporate different types of predictors outperformed those that relied solely on one type of predictor (e.g., clinical or imaging). No consistent patterns of clinical, radiological or radiomics features were identified for each distinct outcome of ER, LR, LTP, and LTPFS.

Preprocedural identification of patients at high risk for ER following TA is currently mostly dependent on diagnostic image evaluation and histopathological examination. However, associated factors such as microvascular invasion or microsatellite nodules often go undetected using these methods, and preprocedural diagnostic biopsies are not standard clinical routine in HCC [34,35,36]. Prognostic modeling offers a non-invasive, individualized risk stratification that may bridge this gap. The externally validated model by Huang et al. [25] performed best to predict ER (AUC-ROC: 0.83). Among other parameters, peri- and intratumoral delta-radiomics features were incorporated, capturing dynamic changes in imaging characteristics across multiple MRI phases acquired prior to treatment. These features were hypothesized to reflect tumor heterogeneity and biological aggressiveness, as a potential substitute for invasive histopathological examination. Exploring the prognostic value of preprocedural intra- and/or peritumoral characterization through multiphasic imaging analysis (i.e., arterial phase, portal venous phase, hepatobiliary phase, diffusion-weighted imaging) was performed in other studies as well, with varying methods: one strategy involved extracting features separately from each imaging phase using radiomics or DL, followed by feature selection (AUC-ROC range: 0.78–0.82) [15,20,26]. Another approach captured changes between phases via signal intensity differences (AUC-ROC range: 0.75–0.84) [19,21,22,24,27]. In a more comprehensive approach, Ma et al. [23] incorporated both spatial and temporal features by using spatiotemporal building blocks (namely Bi-LSTM) in their deep learning model, which automatically learned and integrated features from CEUS data to capture dynamic tumor behavior. Their method demonstrated improved performance (AUC-ROC for ER: 0.84; C-index: 0.77) compared to models that utilized only spatial information from ultrasound imaging.

Inconsistency was found between studies in the definition of ER. This disparity matters, because classifying cases with LTP or residual tumor as an “event” in preprocedural ER prediction assumes that the same prognostic factors apply for both outcomes. In the literature, some overlapping but predominantly different associated factors for each distinct outcome are described [9,10]. Residual tumor detected at the ablation site during the first follow-up scan may be misclassified as either ER or LTP, when in fact it represents incomplete ablation. LTP, by definition, refers to the reappearance of viable tumor at the ablation margin after at least one contrast-enhanced CT scan has confirmed the absence of residual viable tumor following ablation [37]. Non-standardized use of clinical outcomes can lead to inaccurate risk prediction and consequently suboptimal patient management when used in clinical practice. In addition, it reduces the reliability of model comparisons across studies. Therefore, clear and consistent definitions, as described in guidelines on definition of (time-to-event) endpoints [37,38] and international HCC management guidelines [2,39], are essential.

RFS provides a time-based estimate of how long a patient is likely to remain disease-free, enabling tailored postprocedural monitoring. Yet again, inconsistency was found for the definition of RFS with some studies including death as an event despite it being a competing risk in the context of curative-intent treatment for early-stage HCC [19,30], and some excluding it [15,18,20,21] which is more appropriate in this setting [37]. Implementing advanced feature selection methods such as LASSO [18] and DL-based approaches like CNNs [20] showed enhanced predictive performance. Models that relied on a single radiomic feature (tumoral or peritumoral) showed poorer performance compared to those including a combination of features from both regions [29]. The combined model by Li JP et al. [18] showed excellent discriminative and calibration performance (AUC-ROC: 0.96 (95% CI: 0.91–1.00); C-index: 0.92; HL-test: *p* = 0.716). However, since only internally tested models were developed for this outcome, these comparisons offer limited insight. The absence of external testing increases the risk of overfitting, as models may capture dataset-specific patterns rather than generalizable patterns. Consequently, these reported metrics may overestimate the true performance of the models. External testing is therefore needed before these models can be implemented in clinical practice.

The advantage of a per-tumor LTP risk stratification is that it may assist in lesion specific postprocedural monitoring, thereby identifying the need for adjunctive locoregional treatment at an early stage. Two models were developed and externally tested for predicting LTP and LTPFS: Li FY et al. [16] and Chen et al. [17], reporting a C-index of 0.76 and AUC-ROC of 0.87, respectively. The mutual predictive parameter was AM, a parameter shown to be an independent predictor of LTP following TA of HCC in a previous study by Laimer et al. [4]. Furthermore, Li FY et al. [16] measured the AM plane-by-plane (i.e., 2-dimensional), whereas Chen et al. [17] employed 3D reconstruction for analysis. The latter approach is considered to be more accurate, since two-dimensional AM calculations may miss information along the depth axis. To address limitations of AM measurements, such as interobserver reliability and reliance on precise image registration, Chen et al. [17] incorporated advanced multiparametric DL and radiomic features from pre- and post-ablation MRI images in the region of interest, alongside the AM. This model potentially reduces observer dependency and is easier to implement across diverse clinical settings, including those without access to advanced image registration and AM measurement software.

Variability in outcome definitions and methodological quality complicated the identification of best practices for model development techniques and the determination of mutual prognostic variables across outcomes. Nevertheless, analyses of studies that developed multiple models, consistently demonstrated that combining clinical variables with imaging features resulted in better prognostic performance than using either imaging features alone [20,29] or clinical features alone [23]. Comparable findings, supporting the enhanced prognostic performance when combining clinical and imaging variables, have been reported in the literature [7,32,40,41]. At this stage, the reviewed prognostic models are not suitable to be used in clinical practice yet, due to various reasons. First, many models lacked either an independent internal or external test set, both of which are crucial for assessing generalizability. When data availability is limited, techniques like cross-validation and bootstrapping are recommended methods for model validation [42], as pursued by Ma et al. [23] to overcome overfitting. However, some studies [16,26,29] used these techniques for testing, which may have led to overoptimistic performance estimates. We therefore strongly recommend that future studies prioritize on reliable internal testing methods and external testing using an independent cohort. Second, most models were classified as having a high ROB, due to unclarities in patient selection, incomplete reporting on feature selection, blinding during predictor assessment, inadequate clarification of outcome definitions and use of multivariate analysis for predictor selection. To overcome these issues, adherence to the Transparent reporting of a multivariable prediction model for individual prognosis or diagnosis (TRIPOD) checklist is strongly recommended during model development [13,43]. And in addition to this, we advocate the use of standardized terminology, as described in guidelines on definition of (time-to-event) endpoints [37]. Lastly, given the global etiological variability in HCC patients, the applicability of the reviewed models should be considered limited in populations and/or endemic regions on which they were not trained. Over 65% of patients in the included cohorts were infected by hepatitis B and/or C, which may limit the generalizability of these models in countries with other patient characteristics. Should predictive models similar to these be used in the future, it is crucial to carefully assess population characteristics before their implementation in clinical settings.

This study has some limitations, the included studies were highly heterogeneous in terms of predictor types, modeling approaches, outcome definitions, and validation methods, which limited direct comparability and precluded meta-analysis. Additionally, ROB was assessed using PROBAST, which was not specifically developed for AI-based prediction models and may not fully capture biases unique to these techniques. Lastly, this review focused exclusively on recurrence outcomes and did not consider other clinically relevant outcomes, such as post-procedural liver decompensation, despite its influence on prognosis for this patient population.

## 5. Conclusions

Tumor recurrence and LTP are crucial determinants of long-term outcomes in patients with HCC treated with TA. This review revealed variability in model development methodology and incorporated predictors. Models that integrated multiple types of predictors such as clinical and radiological features outperformed those relying solely on one type of predictor. However, prior to integrating these models in clinical practice, future studies must focus on standardizing outcome definitions, testing models in external cohorts, and ensuring transparent and reproducible development methods. Until these challenges are addressed, current evaluated models should be regarded as promising but preliminary tools for individualized risk stratification in the context of TLA.

## Figures and Tables

**Table 1:** Characteristics of included articles in terms of tumor criteria and outcome definition.

Reference	Tumor Related Inclusion Criteria	Outcome	Time Period	Definition of Outcome
Liu et al. (2020) [20]	Single tumor ≤ 5 cm	PFS	TP < 2 years following TA	Time to progression (LTP, new intrahepatic tumor, vascular invasion, or distant organ metastases). LTP: tumor adjacent to ablation margin < 1.0 cm
Li JP et al. (2022) [18]	Single tumor ≤ 3 cm, or sum of 2 tumors ≤ 3 cm	TP	<12 months following TA	Tumor progression: LT-TR viable lesion
Wang R et al. (2023) [21]	Single tumor < 5 cm or ≤3 tumors each ≤ 3 cm	RFS	The interval between the initial date of TLA and the date of the tumor recurrence or last follow-up visit before 1 October 2021.	Tumor recurrence: LR, IDR, and EM
Zhang L et al. (2021) [29]	HCC with longest diameter > 10 mm, without capsular, adjacent organ and/or vascular invasion	RFS	The interval between the initial date of TA and the date of the tumor recurrence	Time to recurrence
Cha et al. (2023) [19]	Single tumor ≤ 3 cm	ER RFS	ER: <2 years following TA RFS: at 1- or 2- or 5-years after RFA.	Recurrence: LTP, IDR, and EM RFS: Time to the development of recurrence or death
Zhang X et al. (2022) [26]	Single tumor < 5 cm, or <3 tumors each < 3 cm	ER	<2 years following TA	New cancerous focus with typical imaging features of the liver or other organs
Ni et al. (2022) [24]	Not specified	ER	<2 years following TA	Recurrence: local and distant IH recurrence. IH local recurrence: active tumors found in adjacent or ablated areas < 1 month of follow-up after ablation. IDR: tumors in the liver parenchyma outside the ablation site on any postprocedural image during the follow-up period
Hu et al. (2021) [22]	≤3 tumors each ≤ 5 cm	ER	<2 years following TA	LTP, IDR and EM
Zhang Z et al. (2022) [28]	Single tumor < 5 cm, or ≤3 tumors each < 3 cm	ER	<2 years following TA	The presence of new IH and/or EH lesions
Huang et al. (2024) [25]	Not specified	RFS ER	RFS: time from the date of operation to the date of the first recurrence ER: <2 years following TA	Recurrence: IH and/or EM
Wang Y et al. (2024) [15]	Single tumor < 5 cm or ≤3 tumors each ≤ 3 cm	IR	<2 years following TA	IH recurrence (local or distant)
Ma et al. (2021) [23]	Single tumor < 5 cm	ER LR risk	ER: <2 years following TA LR: >2 years following TA	LTP, IDR and ER
Wu et al. (2022) [30]	Single tumor < 5 cm, or <3 tumors each < 3 cm	RFS ER LR	RFS: time between the treatment and disease recurrence or death ER: <2 years following TA LR: <5 years following TA	ER: Time to recurrence (excluding LTP) LR: Time to recurrence RFS: Time to recurrence or death
Lv et al. (2021) [27]	Not specified	AIR	>6 months of disease-free status following TA	AIR: Simultaneous development of multiple nodular (>3) or infiltrative recurrence in the treated segment of the liver
Li FY et al. (2021) [16]	Single HCC ≤ 3 cm, without major vascular infiltration or extrahepatic metastasis	LTPFS	Within 6-, 12-, and 24- months following TA	LTPFS: Time from ablation to the date of LTP. LTP: enhancements in lesion in the arterial phase with a washout lesion in the delayed phase of a contrast-enhanced imaging examination (CEUS, CT, or MRI) inside or abutting the ablation area during follow-up.
Chen et al. (2023) [17]	Single tumor <5 cm, or ≤3 tumors < 3 cm	LTP	No predefined time frame. The median follow-up duration for all patients was 22.5 months (IQR, 11.2–55.3 months).	Abnormal nodular, disseminated, and/or unusual patterns of peripheral enhancement around the ablative site on imaging

Abbreviations: AIR, Aggressive intrasegmental recurrence; CEUS, Contrast-enhanced ultrasonography; CT, Computed tomography; EM, Extrahepatic metastasis; ER, Early recurrence; HCC, Hepatocellular carcinoma; IDR, Intrahepatic distance recurrence; IQR, Interquartile range; IR, Intrahepatic recurrence; LR, Local recurrence; LT-TR, Liver imaging reporting and data system (LI-RADS)-treatment response; LTP, Local tumor progression; LTPFS, Local tumor progression free survival; MRI, Magnetic resonance imaging; PFS, Progression free survival; RFS, Recurrence free survival; TA, Thermal ablation; TP, Tumor progression.

**Table 2:** Characteristics of included articles in terms of dataset and evaluation status.

Reference	Data Source	Ablation Technique	Imaging Modality	Sample Size			Test Cohort	
				Training	Validation	Test	Internal	External
Liu et al. (2020) [20]	Single center	RFA	CEUS	149	0	65	+	-
Li JP et al. (2022) [18]	Single center	RFA	DECT	63	0	Model 1:2000 BSR Model 2: 10-Fold CV Model 3: 2000 BSR	+	-
Wang R et al. (2023) [21]	Single center	RFA	GAE-MRI	153	0	51	+	-
Zhang L et al. (2021) [29]	Single center	RFA MWA	GAE-MRI	92	0	1000 BSR	+	-
Cha et al. (2023) [19]	Single center	RFA	GAE-MRI	152	0	1000 BSR	+	-
Zhang X et al. (2022) [26]	Single center	RFA	CEMRI	63	0	27	+	-
Ni et al. (2022) [24]	Single center	RFA	CEUS	60	0	48	+	-
Hu et al. (2021) [22]	Single center	RFA MWA	GAE-MRI	112	0	48	+	-
Zhang Z et al. (2022) [28]	Single center	MWA	CEMRI	226	0	113	+	-
Huang et al. (2024) [25]	Single center	RFA MWA	Gadobenate dimeglumine-MRI	110	0	From different temporal period: 129 From different scanner: 25	+	+
Wang Y et al. (2024) [15]	Multicenter	RFA MWA	CEMRI	335	84	From two different centers: 116	-	+
Ma et al. (2021) [23]	Single center	RFA MWA	Model nr. 1 & 5: CEUS Model nr. 2 & 6: US Models 4 & 8: CEUS/US	255	5-Fold CV	63	+	-
Wu et al. (2022) [30]	Single center	MWA	US	400	0	113	+	-
Lv et al. (2021) [27]	Single center	RFA	CEMRI	40	0	18	+	-
Li FY et al. (2021) [16]	Multicenter	RFA MWA	MRI/CT/CEUS	296	0	148	+	-
Chen et al. (2023) [17]	Multicenter	RFA MWA	CEMRI	151	0	From center 1: 38 From center 2: 135 From center 3: 93	+	+

Abbreviations: BSR, Bootstrapping resampling; CEMRI, Contrast enhanced MRI; CEUS, Contrast enhanced ultrasound; CV, Cross validation; DECT, Dual energy computed tomography; GAE-MRI, Gadoxetic acid enhanced MRI; MRI, Magnetic resonance imaging; MWA, Microwave Ablation; RFA, Radiofrequency Ablation; US, Ultrasound.

**Table 3:** Characteristics of included models in terms of feature selection, model development techniques, and list of predictors.

Reference	Model nr.	Feature Selection Technique	Model Development Technique	Predictors				
				Preprocedural	Postprocedural	EPV	Clinical	Imaging
Liu et al. (2020) [20]	1	Through CNN framework	Cox-CNN proportional hazard model	+	-	NA	None	64-dimensional vector as DL-based features
	2	MV Cox Regression on CNN features	Nomogram via MV Cox regression	+	-	NA	1. Age 2. PLT	1. Tumor size 2. Survival hazard based on radiomics signatures
Li JP et al. (2022) [18]	1	UV and MV logistic regression	MV logistic regression	-	+	2.9	1. ALBI 2. λ_AP(40–100 keV)_	Iodine concentration in the AP within the ROI
	2	LASSO algorithm	Linear regression model	-	+	NI	None	6 Radiomics features from first order statistics (1), GLCM (2), GLDM (3)
	3	Integration of clinical and radiomics features from models nr. 1 and 2	Nomogram via MV logistic regression	-	+	2.5	Features from model nr. 1	Radiomics features from model nr. 2
Wang R et al. (2023) [21]	1	UV and MV logistic regression	Nomogram via Cox proportional hazards regression	+	-	NI	AFP > 100 ng/ml	1. Rim AP hyperenhancement 2.Targetoid restriction on DWI
Zhang L et al. (2021) [29]	1	UV and MV Cox regression	MV Cox regression	+	-	2.4	1. Albumin 2. GGT 3. AFP	Tumor size
	2	1. ICC > 0.75 2. RSF with VIMP-based ranking	Random survival forest	+	-	0.0	None	6 peritumoral (5 mm), 6 peritumoral (5 + 5 mm) and 8 tumoral radiomics features from first order statistics (4), GLCM (9), GLRLM (4), GLDM (1), NGTDM (2).
	3	Integration of features from models nr. 1 and 2	Random survival forest	+	-	1.5	Features from model nr. 1	1. Tumor size 2. Radiomics features from model nr. 2
Cha et al. (2023) [19]	1 2	UV and MV Cox regression	Nomogram via MV Cox regression	+	-	Model 1: 2.7 Model 2: 2.8	1. Age 2. ALBI-grade 3. MoRal score > 68	1. Non-rim AP hyperenhancement 2. Enhancing capsule 3. Low signal intensity on HBP 4. High risk MVI
Zhang X et al. (2022) [26]	1	1. AK native algorithm 2. ICC > 0.75	Logistic regression	+	-	0.0	None	Radiomics features from first order statistics (2), GLCM (3), GLDM (4)
	2	Radiomic: Features from model nr. 1 Clinical and radiological: UV and MV logistic regression	Nomogram via MV logistic regression	+	-	0.0	Albumin level	1. Number of tumors 2. Radiomics features from model nr. 1
Ni et al. (2022) [24]	1	UV and MV logistic regression	Nomogram via MV logistic regression	+	-	1.1	1. Neutrophil-to-lymphocyte ratio 2. AFP	1. Number of tumors 2. CEUS enhancement pattern
Hu et al. (2021) [22]	1	UV and MV logistic regression	Nomogram via MV logistic regression	+	-	7.1	AFP	1. Tumor number 2. Arterial peritumoral enhancement 3. satellite nodule 4. Peritumoral hypo intensity on HBP
Zhang Z et al. (2022) [28]	1	UV and MV logistic regression	Multi variable Cox regression	+	+	7.2	None	1. Tumor size 2. MAM 3. Recurrence score: 3.1. Ill-defined ablation margin 3.2. Capsule enhancement 3.3. ADC 3.4. ∆ADC 3.5. EADC
Huang et al. (2024) [25]	1	UV and MV logistic regression	MV logistic regression	+	-	2.7	Child-Pugh score	1. High-risk tumor location 2. Incomplete or absent tumor capsule
	2	1. ICC ≤ 0.75 2. Pearson CC (threshold > 0.99) 3.ANOVA 4.Logistic Regression	Logistic regression	+	-	0.4	None	12 radiomics features from GLDM (9), GLSZM (1), GLRLM (1), NGTDM (1)
	3	Integration of features from models 1 and 2	Nomogram via MV logistic regression	+	-	3.2	Features from model nr. 1	Features from models nr. 1 and 2
Wang Y et al. (2024) [15]	1	1. ICC < 0.7 2. Decision tree ranking 3. UV Cox proportional hazards	MV Cox regression	+	-	NI	None	15 radiomics features from first order statistics (7), GLCM (3), GLRLM (1), GLSZM (4)
	2	CNN framework	3D-CNN	+	-	NI	None	128-dimensional DL-based feature vector
	3	UV and MV logistic regression	MV logistic regression	+	-	NI	Serum albumin level	1. Number of tumors 2. Features from radiomics and DL models
Ma et al. (2021) [23]	1 5	Through DL framework	DL model	+	-	NA	None	Relevant features selected by DL model
	2	LASSO regression with CV	Logistic regression	+	-	31	None	2 radiomics features from GLDM (1), GLCM (2)
	3	UV and MV logistic regression	MV logistic regression	+	-	31	1. APRI 2. PLT 3. Monocyte	None
	6	LASSO regression with CV	Logistic regression	+	-	13.2	None	4 radiomics features from first order statistics (2), GLDM (1), GLSZM (1)
	7	UV and MV Cox proportional hazards regression	MV Cox proportional hazards	+	-	17.67	1.Portal hypertension 2. ALT 3. Hemoglobin	None
	4 8	Integration of selected features from CEUS, US, and clinical models using logistic regression	Nomogram via MV logistic regression	+	-	Model 4: 18.6 Model 8: 10.6	Same as clinical model	1. DL score 2. Radiomics score
Wu et al. (2022) [30]	1	Clinical and radiological: UV and MV Cox regression US semantic: Correlation analysis	MV Cox regression	+	-	NA	1. AFP 2. ALBI 3. AST 4. TBIL	1. Tumor size 2. Number of tumors 3. US semantic features: 3.1. Echogenicity 3.2. Morphology 3.3. Hypoechoic halo 3.4. Boundary 3.5. Posterior acoustic enhancement 3.6. Intertumoral vascularity
	2	Radiological: UV and MV Cox regression. DL: ResNet18 framework	MV Cox regression	+	-	NA	None	1. Tumor size 2. Number of tumors 3. DL-based features
	3	Clinical and radiological: UV and MV Cox regression. DL: ResNet18 framework	MV Cox regression	+	-	NA	1. AFP 2. PLT	1. Tumor size 2. Number of tumors 3. DL-based features
Lv et al. (2021) [27]	1	LASSO algorithm	MV logistic regression	+	-	0.0	None	2 radiomics features from GLSZM (1), GLRLM (1)
	2	UV and MV logistic regression	MV logistic regression	+	-	2.5	None	1. Tumor shape 2. ADC value 3. DWI signal intensity 4. ΔSI enhancement rate
Li FY et al. (2021) [16]	1	UV and MV Cox regression	Nomogram via MV Cox regression	+	+	2.6	None	1. Tumor size 2. Ablation margin
Chen et al. (2023) [17]	1	UV and MV logistic regression	Support vector machine	+	+	1.9	None	1. Number of tumors 2. Location of abutting major vessels 3. Ablation margin
	2	1. Reliability evaluation 2. UV regression 3. Boruta method	Support vector machine	+	+	<1	None	1. 8 DL-based features 2. 12 radiomics features from first order statistics (3), GLCM (3), NGTDM (2), GLSZM (1), GLRLM (2), shape features (1)
	3	Integration of features from models nr. 1 and 2	Support vector machine	+	+	0.8	None	Features of models 1 and 2

Abbreviations: ADC, Apparent diffusion coefficient; AK, Artificial intelligence kit from GE Healthcare; ALBI, Albumin-bilirubin grade; ALT, Alanine aminotransferase; AFP, Alpha fetoprotein; AP, Arterial phase; APRI, (AST/40) × 100/PLT; AST, Aspartate aminotransferase; CNN, Convolutional neural network; DL, Deep learning; DWI, Diffusion weighted imaging; EADC, Exponential apparent diffusion coefficient; GGC, Gamma-glutamyl transferase; GLDM, Gray level dependence matrix; GLCM, Gray level co-occurrence matrix; GLRLM, gray level run length matrix; GLSZM, Gray level size zone matrix; HBP, Hepato-biliary phases; ICC, Interclass correlation coefficient; MAM, Minimal ablation margin; MoRal, Model Of Recurrence After Liver transplant; MV, Multivariate; MVI, Microvascular invasion; NA, Not available; NI, No information; NGTDM, Neighborhood gray tone difference matrix; Nr, number; PLT, Platelet count; RFS, Random survival forest; TBIL, Total bilirubin; UV, Univariate.

**Table 4:** Characteristics of included models in terms of predictor types and performance.

Paper	Nr.	Modeling		Predictors				Out Come	AUC-ROC	C-Index	Kaplan–Meier	Calibration	DCA	Cohort
		AI	C	Cl	R	RM	DL							
Liu et al. [20]	1.							RFS *	0.81 (0.72–0.93) ^††^	0.73 (0.65–0.80)	*p* < 0.005	-	+	IV
	2.							RFS *	0.82 (0.72–0.93) ^††^	0.73 (0.68–0.84)	*p* < 0.005	HL *p* = 0.479	ThP: >30%	IV
Li JP et al. [18]	1.							RFS *	0.92 (0.85–0.99) ^†^	0.92	-	HL *p* = 0.792	+	IV
	2.							RFS *	0.90 (0.82–1.00) ^†^	-	-	-	+	IV
	3.							RFS *	0.96 (0.91–1.00) ^†^	0.96	-	HL *p* = 0.71	+	IV
Wang R et al. [21]	1.							RFS	-	0.81 (0.71–0.90)	-	+	+	IV
Zhang L et al. [29]	1.							RFS	0.67 ^†^*	0.70 (0.64–0.76)	-	-	+	IV
	2.							RFS	0.82 ^†^*	0.61 (0.50–0.70)	-	-	+	IV
	3.							RFS	0.83 ^†^*	0.71 (0.64–0.76)	*p* = 0.007	-	+	IV
Cha et al. [19]	1.							RFS	-	0.72 (0.66–0.77)	*p* < 0.001	-	-	IV
	2.							ER	-	0.75 (0.68–0.81)	*p* < 0.001	-	-	IV
Zhang X et al. [26]	1.							ER	0.81	-	-	-	-	IV
	2.							ER	0.81	-	-	+	+	IV
Ni et al. [24]	1.							ER	0.78	-	-	+	ThP: 4.3–87.3%	IV
Hu et al. [22]	1.							ER	0.83 (0.71–0.96)	0.75	*p* < 0.001	HL *p* = 0.168	ThP: 24–99%	IV
Zhang Z et al. [28]	1.							ER	0.85 (0.71–0.87)		*p* < 0.05	-	-	IV
Huang et al. [25]	1.							ER	0.77 (0.55–0.91)	-	-	-	+	EV
	2.							ER	0.77 (0.56–0.91)	-	*p* < 0.0001	-	+	EV
	3.							ER	0.83 (0.62–0.95)	-	*p* < 0.0001	HL *p* = 0.397	+	EV
Wang Y et al. [15]	1.							IHER	0.75 (0.64–0.75)	-	-	-	+	EV
	2.							IHER	0.64 (0.51–0.65)	-	-	-	+	EV
	3.							IHER	0.79 (0.67–0.83)	-	-	-	+	EV
Ma et al. [23]	1.							ER	0.83 (0.81–0.85)	-	-	-	-	IV
	2.							ER	0.56 (0.53–0.59)	-	-	-	-	IV
	3.							ER	0.60 (0.57–0.63)	-	-	-	-	IV
	4.							ER	0.84 (0.82–0.86)	-	-	+	+	IV
	5.							LR	-	0.68 (0.65–0.71)	*p* < 0.0001	-	-	IV
	6.							LR	-	0.56 (0.53–0.59)	*p* = 0.08	-	-	IV
	7.							LR	-	0.67 (0.64–0.70)	*p* < 0.0001	-	-	IV
	8.							LR	-	0.77 (0.76–0.78)	*p* < 0.0001	+	+	IV
Wu et al. [30]	1.							ER	-	0.70 (0.64–0.84)	*p* < 0.001	+	-	IV
	2.							LR	-	0.72 (0.62–0.80)	*p* < 0.001	+	-	IV
	3.							RFS	-	0.72 (0.65–0.79)	*p* < 0.001	+	-	IV
Lv et al. [27]	1.							AIR	0.64 (0.34–0.93)	-	-	+	ThP: >8%	IV
	2.							AIR	0.82 (0.58–1.00)	-	-	+	+	IV
Li FY et al. [16]	1.							LTPFS	-	0.76 (0.62–0.89)	*p* = 0.001	+	-	IV
Chen et al. [17]	1.							LTP	0.80 (0.72–0.89)	-	-	-	-	EV
	2.							LTP	0.86 (0.80–0.91)	-	-	-	-	EV
	3.							LTP	0.87 (0.82–0.91)	-	*p* = 0.0021	+	-	EV

Abbreviations: AIR, Aggressive Intrasegmental Recurrence; AI, Artificial Intelligence based statistics; AUC-ROC, Area Under the Curve of the Receiver Operator characteristic Curve; C, Conventional statistics; Cl, Clinical; C-index, Concordance-Index; DCA, Decision Curve Analysis; DL, Deep Learning; ER, Early Recurrence; EV, External Validation; HL, Hosmer Lemeshow test; IHER, Intrahepatic Early Recurrence; IV, Internal Validation; LR, Late Recurrence; LTP, Local Tumor Progression; LTPFS, Local Tumor Progression Free Survival; Nr, number; R, Radiological; RFS, Recurrence Free Survival; RM, Radiomics; TP, Threshold Probability. * In the original article formulated as PFS, according to standard terminology reformulated as RFS. ^†^ At 12 months ^††^ At 24 months ^†^* At fixed time points of 12 to 70 months. Color code: dark orange—AI; light orange—conventional; gray—clinical; blue—radiological; pink—radiomics; yellow—deep learning.

## Data Availability

Data extraction forms are available upon request.

## Abbreviations

The following abbreviations are used in this manuscript:

AI	Artificial intelligence
AFP	Alpha fetoprotein
ALBI	Albumin-bilirubin
AUC-ROC	Area under the receiver operating characteristic curve
BCLC	Barcelona Clinic Liver Cancer
CEUS	Contrast-enhanced ultrasound
CNN	Convolutional neural network
CI	Confidence interval
C-index	Concordance index
DL	Deep learning
EPV	Event per variable
ER	Early recurrence
HCC	Hepatocellular carcinoma
HL	Hosmer–Lemeshow
LI-RADS	Liver Imaging Reporting and Data System
LTP	Local tumor progression
LTPFS	Local tumor progression free survival
LR	Late recurrence
MWA	Microwave ablation
MV	Multivariate
PRISMA	Preferred reporting items for systematic reviews and meta-analysis
PROSPERO	International prospective register of systematic review
RFA	Radiofrequency ablation
RFS	Recurrence free survival
ROB	Risk of bias
TA	Thermal ablation
UV	Univariate

## Appendix A

**Table A1:** Search strategy used for PubMed, Web of science, Embase and Cochrane.

Database	Search	Hits
Pubmed	(((thermoablat*[tiab] OR “RFA”[tiab] OR “MWA”[tiab] OR ((“thermo”[tiab] OR “thermal”[tiab] OR “radiofrequenc*”[tiab] OR “Microwaves”[Majr] OR “Microwave*”[tiab]) AND (“Ablation techniques”[Mesh] OR “Radiofrequency Ablation”[Majr] OR “ablat*”[tiab]))) AND (“Liver Neoplasms”[Mesh] OR HCC[tiab] OR ((“liver”[tiab] OR “livers”[tiab] OR “hepatic”[tiab] OR hepatocellular[tiab]) AND (“neoplasms”[tiab] OR “neoplasm”[tiab] OR “cancer”[tiab] OR “cancers”[tiab] OR “tumor”[tiab] OR “tumors”[tiab] OR “tumour”[tiab] OR “tumours”[tiab] OR “malignan*”[tiab] OR “carcinom*”[tiab]))) AND ((“Nomograms”[Mesh] OR nomogram*[tiab] OR nomograph*[tiab] OR “prognostic model*”[tiab] OR “predictive model*”[tiab] OR “prediction model*”[tiab] OR ((“prognos*”[ti] OR “predict*”[ti]) AND model*[ti])) OR ((“prognos*”[ti] OR “predict*”[ti] OR “prognosis”[majr]) AND (“Artificial Intelligence”[Mesh] OR “AI”[ti] OR “artificial intelligen*”[tiab] OR “AI”[tiab] OR “machine learn*”[tiab] OR “deep learn*”[tiab] OR “neural network*”[tiab] OR “support vector machine*”[tiab] OR “reinforcement learning”[tiab] OR “Markov”[tiab] OR “decision tree*”[tiab] OR “random forest”[tiab] OR “Bayesian network*”[tiab] OR “convolutional network”[tiab] OR “radiomic*”[tiab] OR “gradient boost*” [tiab] OR “feature selection*”[tiab])))) OR ((thermoablat*[tiab] OR “RFA”[tiab] OR “MWA”[tiab] OR ((“thermo”[tiab] OR “thermal”[tiab] OR “radiofrequenc*”[tiab] OR “Microwaves”[Majr] OR “Microwave*”[tiab]) AND (“Ablation techniques”[Mesh] OR “Radiofrequency Ablation”[Majr] OR “ablat*”[tiab]))) AND (“Carcinoma, Hepatocellular”[Majr] OR “HCC”[tiab] OR “hepatocellular carcinoma*”[tiab]) AND (“primary”[tiab]) AND ((“Nomograms”[Mesh] OR nomogram*[tiab] OR nomograph*[tiab] OR “prognostic model*”[tiab] OR “predictive model*”[tiab] OR “prediction model*”[tiab] OR ((“prognos*”[ti] OR “predict*”[ti]) AND model*[ti])) OR (((“prognos*”[ti] OR “predict*”[ti] OR “prognosis”[majr]) AND (“Artificial Intelligence”[Mesh] OR “AI”[ti] OR “artificial intelligen*”[tiab] OR “AI”[tiab] OR “machine learn*”[tiab] OR “deep learn*”[tiab] OR “neural network*”[tiab] OR “support vector machine*”[tiab] OR “reinforcement learning”[tiab] OR “Markov”[tiab] OR “decision tree*”[tiab] OR “random forest”[tiab] OR “Bayesian network*”[tiab] OR “convolutional network”[tiab] OR “radiomic*”[tiab] OR “gradient boost*”[tiab] OR “feature selection*” [tiab])))))) OR ((thermoablat*[tiab] OR “RFA”[tiab] OR “MWA”[tiab] OR ((“thermo”[tiab] OR “thermal”[tiab] OR “radiofrequenc*”[tiab] OR “Microwaves”[Mesh] OR “Microwave*”[tiab]) AND (“Ablation techniques”[Mesh] OR “Radiofrequency Ablation”[Mesh] OR “ablat*”[tiab]))) AND ((“Liver Neoplasms”[Mesh] OR ((“liver”[tiab] OR “livers”[tiab] OR “hepatic”[tiab]) AND (“neoplasms”[tiab] OR “neoplasm”[tiab] OR “cancer”[tiab] OR “cancers”[tiab] OR “tumor”[tiab] OR “tumors”[tiab] OR “tumour”[tiab] OR “tumours”[tiab] OR “malignan*”[tiab] OR “carcinom*”[tiab]))) OR (“primary”[tiab] OR “Carcinoma, Hepatocellular”[Mesh] OR “HCC”[tiab] OR “hepatocellular carcinoma*”[tiab])))) AND (“Artificial Intelligence”[Mesh] OR “AI”[ti] OR “artificial intelligen*”[tiab] OR “AI”[tiab] OR “machine learn*”[tiab] OR “deep learn*”[tiab] OR “neural network*”[tiab] OR “support vector machine*”[tiab] OR “reinforcement learning”[tiab] OR “Markov”[tiab] OR “decision tree*”[tiab] OR “random forest”[tiab] OR “Bayesian network*”[tiab] OR “convolutional network”[tiab] OR “radiomic*”[tiab] OR “gradient boost*”[tiab] OR “feature selection*”[tiab]))	353
WebScience	((((thermoablat* OR “RFA” OR “MWA” OR ((“thermo” OR “thermal” OR radiofrequenc* OR Microwave*) AND (ablat*))) AND (HCC OR ((“liver” OR “livers” OR “hepatic” OR hepatocellular) AND (“neoplasms” OR “neoplasm” OR “cancer” OR “cancers” OR “tumor” OR “tumors” OR “tumour” OR “tumours” OR malignan* OR carcinom*))) AND ((nomogram* OR nomograph* OR “prognostic model” OR “prognostic models” OR “predictive model” OR “predictive model” OR “prediction model” OR “prediction models” OR ((prognos* OR predict*) AND model*)) OR ((prognos* OR predict*) AND (“artificial intelligence” OR “AI” OR “machine learning” OR “deep learning” OR “neural network” OR “neural networks” OR “Machine Intelligence” OR “transfer learning” OR “support vector machine” OR “support vector machines” OR “reinforcement learning” OR “Markov” OR “decision tree” OR “decision trees” OR “random forest” OR “Bayesian network” OR “Bayesian networks” OR “convolutional network” OR “convolutional networks”)))) OR ((thermoablat* OR “RFA” OR “MWA” OR ((“thermo” OR “thermal” OR radiofrequenc* OR Microwave*) AND (ablat*))) AND (“HCC” OR “hepatocellular carcinoma” OR “hepatocellular carcinomas”) AND (“primary”) AND ((nomogram* OR nomograph* OR “prognostic model” OR “prognostic models” OR “predictive model” OR “predictive models” OR “prediction model” OR “prediction models” OR ((prognos* OR predict*) AND model*)) OR (((prognos* OR predict*) AND (“artificial intelligence” OR “AI” OR “machine learning” OR “deep learning” OR “neural network” OR “neural networks” OR “Machine Intelligence” OR “transfer learning” OR “support vector machine” OR “support vector machines” OR “reinforcement learning” OR “Markov” OR “decision tree” OR “decision trees” OR “random forest” OR “Bayesian network” OR “Bayesian network” OR “convolutional network” OR “convolutional networks”)))))) OR ((thermoablat* OR “RFA” OR “MWA” OR ((“thermo” OR “thermal” OR radiofrequenc* OR Microwave*) AND (ablat*))) AND ((((“liver” OR “livers” OR “hepatic”) AND (“neoplasms” OR “neoplasm” OR “cancer” OR “cancers” OR “tumor” OR “tumors” OR “tumour” OR “tumours” OR malignan* OR carcinom*))) OR (“primary” OR “HCC” OR “hepatocellular carcinoma” OR “hepatocellular carcinomas”))) AND (“artificial intelligence” OR “AI” OR “machine learning” OR “deep learning” OR “neural network” OR “neural networks” OR “Machine Intelligence” OR “transfer learning” OR “support vector machine” OR “support vector machines” OR “reinforcement learning” OR “Markov” OR “decision tree” OR “decision trees” OR “random forest” OR “Bayesian network” OR “Bayesian networks” OR “convolutional network” OR “convolutional networks”)))	130
Embase	(((thermoablat*.ti,ab. OR “RFA”.ti,ab. OR “MWA”.ti,ab. OR ((“thermo”.ti,ab. OR “thermal”.ti,ab. OR “radiofrequenc*”.ti,ab. OR exp *microwave radiation/ OR “Microwave*”.ti,ab.) AND (exp *radiofrequency ablation/ OR “ablat*”.ti,ab.))) AND (exp liver tumor/ OR HCC.ti,ab. OR ((“liver”.ti,ab. OR “livers”.ti,ab. OR “hepatic”.ti,ab. OR hepatocellular.ti,ab.) AND (“neoplasms”.ti,ab. OR “neoplasm”.ti,ab. OR “cancer”.ti,ab. OR “cancers”.ti,ab. OR “tumor”.ti,ab. OR “tumors”.ti,ab. OR “tumour”.ti,ab. OR “tumours”.ti,ab. OR “malignan*”.ti,ab. OR “carcinom*”.ti,ab.))) AND ((exp nomogram/ OR nomogram*.ti,ab. OR nomograph*.ti,ab. OR “prognostic model*”.ti,ab. OR “predictive model*”.ti,ab. OR “prediction model*”.ti,ab. OR ((“prognos*”.ti. OR “predict*”.ti.) AND model*.ti.)) OR ((“prognos*”.ti. OR “predict*”.ti. OR exp *prognosis/) AND (exp artificial intelligence/ OR “artificial intelligen*”.ti,ab. OR “AI”.ti,ab. OR “machine learn*”.ti,ab. OR “deep learn*”.ti,ab. OR “neural network*”.ti,ab. OR “support vector machine*”.ti,ab. OR “reinforcement learning”.ti,ab. OR “Markov”.ti,ab. OR “decision tree*”.ti,ab. OR “random forest”.ti,ab. OR “Bayesian network*”.ti,ab. OR “convolutional network”.ti,ab. OR “radiomic*”.ti,ab. OR “gradient boost*”.ti,ab. OR “feature selection*”.ti,ab.)))) OR ((thermoablat*.ti,ab. OR “RFA”.ti,ab. OR “MWA”.ti,ab. OR ((“thermo”.ti,ab. OR “thermal”.ti,ab. OR “radiofrequenc*”.ti,ab. OR exp *microwave radiation/ OR “Microwave*”.ti,ab.) AND (exp *radiofrequency ablation/ OR “ablat*”.ti,ab.))) AND (exp *hepatocellular carcinoma cell line/ OR exp *fibrolamellar hepatocellular carcinoma/ OR “HCC”.ti,ab. OR “hepatocellular carcinoma*”.ti,ab.) AND (primary”.ti,ab.) AND ((exp nomogram/ OR nomogram*.ti,ab. OR nomograph*.ti,ab. OR “prognostic model*”.ti,ab. OR “predictive model*”.ti,ab. OR “prediction model*”.ti,ab. OR ((“prognos*”.ti. OR “predict*”.ti.) AND model*.ti.)) OR (((“prognos*”.ti. OR “predict*”.ti. OR exp *prognosis/) AND (exp artificial intelligence/ OR “AI”.ti. OR “artificial intelligen*”.ti,ab. OR “AI”.ti,ab. OR “machine learn*”.ti,ab. OR “deep learn*”.ti,ab. OR “neural network*”.ti,ab. OR “support vector machine*”.ti,ab. OR “reinforcement learning”.ti,ab. OR “Markov”.ti,ab. OR “decision tree*”.ti,ab. OR “random forest”.ti,ab. OR “Bayesian network*”.ti,ab. OR “convolutional network”.ti,ab. OR “radiomic*”.ti,ab. OR “gradient boost*”.ti,ab. OR “feature selection*”.ti,ab.)))))) OR ((thermoablat*.ti,ab. OR “RFA”.ti,ab. OR “MWA”.ti,ab. OR ((“thermo”.ti,ab. OR “thermal”.ti,ab. OR “radiofrequenc*”.ti,ab. OR exp microwave radiation/ OR “Microwave*”.ti,ab.) AND (exp radiofrequency ablation/ OR “ablat*”.ti,ab.))) AND ((exp liver tumor/ OR ((“liver”.ti,ab. OR “livers”.ti,ab. OR “hepatic”.ti,ab.) AND (“neoplasms”.ti,ab. OR “neoplasm”.ti,ab. OR “cancer”.ti,ab. OR “cancers”.ti,ab. OR “tumor”.ti,ab. OR “tumors”.ti,ab. OR “tumour”.ti,ab. OR “tumours”.ti,ab. OR “malignan*”.ti,ab. OR “carcinom*”.ti,ab.))) OR (“primary”.ti,ab. OR exp hepatocellular carcinoma cell line/ OR exp fibrolamellar hepatocellular carcinoma/OR “HCC”.ti,ab. OR “hepatocellular carcinoma*”.ti,ab. AND (“neoplasms”.ti,ab. OR “neoplasm”.ti,ab. OR “cancer”.ti,ab. OR “cancers”.ti,ab. OR “tumor”.ti,ab. OR “tumors”.ti,ab. OR “tumou]r”.ti,ab. OR “tumours”.ti,ab. OR “malignan*”.ti,ab. OR “carcinom*”.ti,ab.))) AND (exp Artificial Intelligence/ OR “AI”.ti. OR “artificial intelligen*”.ti,ab. OR “AI”.ti,ab. OR “machine learn*”.ti,ab. OR “deep learn*”.ti,ab. OR “neural network*”.ti,ab. OR “support vector machine*”.ti,ab. OR “reinforcement learning”.ti,ab. OR “Markov”.ti,ab. OR “decision tree*”.ti,ab. OR “random forest”.ti,ab. OR “Bayesian network*”.ti,ab. OR “convolutional network”.ti,ab. OR “radiomic*”.ti,ab. OR “gradient boost*”.ti,ab. OR “feature selection*”.ti,ab.))	426
Cochrane	(((thermoablat* OR “RFA” OR “MWA” OR ((“thermo” OR “thermal” OR radiofrequenc* OR Microwave*) AND (ablat*))) AND (HCC OR ((“liver” OR “livers” OR “hepatic” OR hepatocellular) AND (“neoplasms” OR “neoplasm” OR “cancer” OR “cancers” OR “tumor” OR “tumors” OR “tumour” OR “tumours” OR malignan* OR carcinom*))) AND ((nomogram* OR nomograph* OR “prognostic model” OR “prognostic models” OR “predictive model” OR “predictive model” OR “prediction model” OR “prediction models” OR ((prognos* OR predict*) AND model*)) OR ((prognos* OR predict*) AND (“artificial intelligence” OR “AI” OR “machine learning” OR “deep learning” OR “neural network” OR “neural networks” OR “Machine Intelligence” OR “transfer learning” OR “support vector machine” OR “support vector machines” OR “reinforcement learning” OR “Markov” OR “decision tree” OR “decision trees” OR “random forest” OR “Bayesian network” OR “Bayesian networks” OR “convolutional network” OR “convolutional networks”)))) OR ((thermoablat* OR “RFA” OR “MWA” OR ((“thermo” OR “thermal” OR radiofrequenc* OR Microwave*) AND (ablat*))) AND (“HCC” OR “hepatocellular carcinoma” OR “hepatocellular carcinomas”) AND (“primary”) AND ((nomogram* OR nomograph* OR “prognostic model” OR “prognostic models” OR “predictive model” OR “predictive models” OR “prediction model” OR “prediction models” OR ((prognos* OR predict*) AND model*)) OR (((prognos* OR predict*) AND (“artificial intelligence” OR “AI” OR “machine learning” OR “deep learning” OR “neural network” OR “neural networks” OR “Machine Intelligence” OR “transfer learning” OR “support vector machine” OR “support vector machines” OR “reinforcement learning” OR “Markov” OR “decision tree” OR “decision trees” OR “random forest” OR “Bayesian network” OR “Bayesian network” OR “convolutional network” OR “convolutional networks”)))))) OR ((thermoablat* OR “RFA” OR “MWA” OR ((“thermo” OR “thermal” OR radiofrequenc* OR Microwave*) AND (ablat*))) AND ((((“liver” OR “livers” OR “hepatic”) AND (“neoplasms” OR “neoplasm” OR “cancer” OR “cancers” OR “tumor” OR “tumors” OR “tumour” OR “tumours” OR malignan* OR carcinom*))) OR (“primary” OR “HCC” OR “hepatocellular carcinoma” OR “hepatocellular carcinomas”)AND (“neoplasms” OR “neoplasm” OR “cancer” OR “cancers” OR “tumor” OR “tumors” OR “tumour” OR “tumours” OR malignan* OR carcinom*))) AND (“artificial intelligence” OR “AI” OR “machine learning” OR “deep learning” OR “neural network” OR “neural networks” OR “Machine Intelligence” OR “transfer learning” OR “support vector machine” OR “support vector machines” OR “reinforcement learning” OR “Markov” OR “decision tree” OR “decision trees” OR “random forest” OR “Bayesian network” OR “Bayesian networks” OR “convolutional network” OR “convolutional networks”))	82

**Table A2:** List of radiomics features included in prognostic models.

Reference	Radiomic Feature	Category						
		First Order Statistics	GLCM	GLDM	GLSZM	GLRLM	NGTDM	Shape Features
Chen et al. [17]	1. original_firstorder_Variance (T1W+C-Post)							
	2. wavelet-LLH_glcm_DependenceEntropy (T1W+C-Pre Extended)							
	3. wavelet-LLH_ngtdm_Coarseness (DWI-Pre)							
	4. wavelet-LLH_firstorder_Entropy (T1W+C-Post-Indented)							
	5. log-sigma-2-0-mm-3D_glszm_ZonePercentage (T1W+C-Pre)							
	6. wavelet-HHL_glcm_JointAverage (T1W+C-Pre)							
	7. wavelet-HHL_glcm_Idmn (T1W+C-Post)							
	8. wavelet-LLH_ngtdm_Coarseness (T2W-Pre-Extended)							
	9. original_shape_Elongation (DWI-Pre)							
	10. wavelet-LLH_glrm_LongRunHighGrayLevelEmphasis (T1W+C-Post)							
	11. wavelet-LLH_glrm_SmallDependenceLowGrayLevelEmphasis (T1W+C-Post)							
	12. wavelet-HHL_firstorder_RobustMeanAbsoluteDeviation (DWI-Pre)							
Li JP et al. [18]	1. Median							
	2. correlation							
	3. sum squares							
	4. large dependence emphasis							
	5. large dependence high gray level emphasis							
	6. large dependence low gray level emphasis							
Zhang X et al. [26]	1. lbp-3D-m2_firstorder_InterquartileRange (AP)							
	2. lbp-3D-k_gldm_DependenceVariance (AP)							
	3. lbp-3D-k_gldm_ShortRunLowGrayLevelEmphasis (T1lbp-3D)							
	4. k_gldm_ShortRunLowGrayLevelEmphasis (DP- WI)							
	5. lbp-3D-m2_glcm_ClusterShade (AP)							
	6. lbp-3D-m2_firstorder_10Percentile (DP)							
	7. wavelet-HLL_glcm_RunEntropy (AP)							
	8. lbp-3D-m1_gldm_LargeDependenceLowGrayLevelEmphasis (AP)							
	9. wavelet-HLL_glcm_DifferenceEntropy (T1WI)							
Huang et al. [25]	1. Dependence Variance (PVP-t1)							
	2. Large Dependence Emphasis(PVP-t1)							
	3. Large Area Low Gray Level Emphasis (PVP-t1)							
	4. Dependence Variance (PVP-AP)							
	5. Dependence Variance (DP-t1)							
	6. Large Dependence Emphasis (DP-t1)							
	7. Run Variance (DP-t1)							
	8. Dependence Variance (DP-t1)							
	9. Dependence Non Uniformity Normalized (DP-AP)							
	10. Dependence Variance (DP-AP)							
	11. Large Dependence Emphasis (DP-AP)							
	12. Coarseness (DP-AP)							
Ma et al. [23]	ER:							
	1. t_wavelet-HLH_gldm_LowGrayLevelEmphasis							
	2. t_wavelet-HHL_glcm_MaximumProbability							
	3. t_wavelet-LLH_glcm_InverseVariance							
	LR:							
	1. t_wavelet-LHH_firstorder_Range							
	2. t_square_gldm_LargeDependenceLowGrayLevelEmphasis							
	3. t_wavelet-HLL_glszm_SizeZoneNonUniformity							
	4. t_squareroot_firstorder_Median							
Zhang L et al. [29]	Peritumoral (5 mm):							
	1. V_wavelet.HLH_firstorder_Kurtosis							
	2.T1_original_glcm_InverseVariance							
	3. T2_wavelet.HHL_firstorder_Skewness							
	4. T1_gradient_glrlm_LongRunHighGrayLevelEmphasis							
	5. T1_gradient_glrlm_ShortRunLowGrayLevelEmphasis							
	6. HBP_squareroot_glcm_InverseVariance							
	Peritumoral (5 mm + 5 mm)							
	7. A_gradient_gldm_DependenceNonUniformityNormalized							
	8. T1_square_glcm_InverseVariance							
	9. T1_gradient_glrlm_ShortRunLowGrayLevelEmphasis							
	10. A_wavelet.HHL_ngtdm_Contrast							
	11. V_wavelet.HLH_firstorder_Kurtosis							
	12. V_wavelet.HHH_glcm_Imc							
	Tumoral:							
	13. T1_original_glcm_InverseVariance							
	14. T2_wavelet.HLH_glcm_SumAverage							
	15. T2_wavelet.HLH_glcm_JointAverage							
	16. T1_wavelet.HHH_glcm_MaximumProbability							
	17. V_wavelet.HLH_firstorder_Skewness							
	18. A_wavelet.LLH_ngtdm_Contrast							
	19. T1_gradient_glrlm_ShortRunLowGrayLevelEmphasis							
	20. V_wavelet.HHH_glcm_MCC							
Wang Y et al. [15]	1. log.sigma.1.0.mm.3D_glcm_InverseVariancer (AC)							
	2. wavelet.HLL_glcm_MCC (AC)							
	3. wavelet.LHL_glrlm_LongRunHighGrayLevelEmphasis (AC)							
	4. log.sigma.4.0.mm.3D_glszm_SmallAreaLowGrayLevelEmphasis (VC)							
	5. wavelet.LHH_glszm_ZoneEntropy (VC)							
	6. log.sigma.3.0.mm.3D_firstorder_Skewness (T2)							
	7. wavelet.LHH_firstorder_RootMeanSquared (T2)							
	8. wavelet.LLH_glszm_SmallAreaLowGrayLevelEmphasis (DC)							
	9. wavelet.HLH_firstorder_Mean (DC)							
	10 wavelet.HHH_glszm_SmallAreaLowGrayLevelEmphasis (DC)							
	11. wavelet.HLL_firstorder_Skewness (DC)							
	12. wavelet.HHH_glcm_Imc1 (FLEX)							
	13.log.sigma.1.0.mm.3D_firstorder_Skewness (FLEX)							
	14. wavelet.HHL_firstorder_Mean (FLEX)							
	15. wavelet.LHH_firstorder_Median (FLEX)							
Lv et al. [27]	1. LowInsentiy-LareAreaEmphasis							
	2. RunLengthNonuniformi ty_AllDirection_offset1_SD							

Abbreviations: GLCM, Gray Level Co-occurrence Matrix features; GLDM, Gray Level Dependence Matrix features; GLSZM, Gray Level Size Zone Matrix features; GLRLM, Gray Level Run Length Matrix features; NGTDM, Neighboring Gray Tone Difference Matrix features. Color code: Gray—First Order Statistics; Orange—GLCM; Yellow—GLDM; Green—GLSZM; Red—GLRLM; Blue—NGTDM; Black—Shape features.

**Table A3:** Risk of bias and applicability assessment for included prognostic models.

Author Year	Model Number	Risk of Bias				Applicability			Overall	
		1.Participants	2. Predictors	3. Outcome	4. Analysis	1.Participants	2. Predictors	3. Outcome	Risk of Bias	Applicability
Liu et al. [20]	1	+	+	+	+	?	+	+	+	?
	2	+	+	+	-	?	+	+	-	?
Li JP et al. [18]	1	+	?	+	-	+	+	+	-	+
	2	+	+	+	-	+	+	+	-	+
	3	+	+	+	-	+	+	+	-	+
Wang R et al. [21]	1	+	?	+	-	+	+	+	-	+
Zhang L et al. [29]	1	+	?	+	-	?	+	+	-	?
	2	+	?	+	-	+	+	+	-	+
	3	+	?	+	-	?	+	+	-	?
Cha et al. [19]	1	+	?	+	-	+	+	+	-	+
	2	+	?	+	-	+	+	+	-	+
Zhang X et al. [26]	1	+	?	+	-	?	+	+	-	?
	2	+	?	+	-	?	+	+	-	?
Ni et al. [24]	1	+	?	+	-	+	+	+	-	+
Hu et al. [22]	1	+	+	+	-	?	+	+	-	?
Zhang Z et al. [28]	1	?	+	+	-	+	+	+	-	+
Huang et al. [25]	1	+	+	+	-	+	+	+	-	+
	2	+	+	+	-	?	+	+	-	?
	3	+	+	+	-	+	+	+	-	+
Wang Y et al. [15]	1	+	?	?	-	?	+	+	-	?
	2	+	?	?	?	?	+	+	?	?
	3	+	-	?	-	+	+	+	-	+
Ma et al. [23]	1	+	?	+	-	?	+	+	-	?
	2	+	?	+	-	?	+	+	-	?
	3	+	?	+	-	?	+	+	-	?
	4	+	?	+	-	?	+	+	-	?
	5	+	?	+	-	?	+	+	-	?
	6	+	?	+	-	?	+	+	-	?
	7	+	?	+	-	?	+	+	-	?
	8	+	?	+	-	+	+	+	-	+
Wu et al. [30]	1	-	?	+	-	?	+	+	-	?
	2	-	?	+	-	?	+	+	-	?
	3	-	?	+	-	?	+	+	-	?
Lv et al. [27]	1	?	?	?	-	?	+	+	-	?
	2	?	?	?	-	?	+	+	-	?
Li FY et al. [16]	1	?	?	+	-	?	+	+	-	?
Chen et al. [17]	1	+	?	+	-	?	+	+	-	?
	2	+	?	+	-	?	+	+	-	?
	3	+	?	+	-	+	+	+	-	+

Legend: Risk of bias: “+” = low risk; “-” = high risk; “?” = unclear risk. Applicability: “+” = low concern; “-” = high concern; “?” = unclear concern.
